# Integrative Network Toxicology, Machine Learning, Single-Cell Analysis, scTenifoldKnk-Based Virtual Knockout, and Molecular Docking Suggest a Potential Molecular Link Between Aspartame and Rheumatoid Arthritis Involving *HLA-DRB1*

**DOI:** 10.3390/ijms27135798

**Published:** 2026-06-26

**Authors:** Tianxi Yan, Qiqi He, Xueli Shi

**Affiliations:** Zhuang Medicine College, Guangxi University of Chinese Medicine, Nanning 530001, China; yantianxi2025@stu.gxtcmu.edu.cn (T.Y.); heqiqi2025@stu.gxtcmu.edu.cn (Q.H.)

**Keywords:** rheumatoid arthritis, aspartame, *HLA-DRB1*, network toxicology, machine learning

## Abstract

Aspartame is a widely used artificial sweetener, but its possible relationship with rheumatoid arthritis (RA) remains insufficiently understood. This study aimed to explore, rather than prove, potential molecular links between aspartame-related targets and RA-associated gene networks. Three public RA transcriptomic datasets (GSE55235, GSE55457, and GSE77298) from the Gene Expression Omnibus (GEO) database were integrated as discovery/training data. Because these datasets included different tissue origins, batch correction was used to reduce dataset-level technical variation, whereas tissue-origin-related biological variation was not assumed to be fully removable. After differential expression analysis, RA-associated differentially expressed genes (DEGs) were identified. The single-cell dataset GSE200815 was used for cell annotation and cellular expression visualization; because its comparator group consists of psoriatic arthritis (PsA) samples rather than healthy controls, single-cell results were interpreted as RA-vs-PsA observations and were not treated as disease-versus-healthy-control evidence. Potential targets of aspartame were retrieved from ChEMBL, SwissTargetPrediction, and the Similarity Ensemble Approach (SEA), and were intersected with RA-related DEGs to construct an aspartame-gene-RA regulatory network. Diagnostic models were developed using 113 machine-learning algorithm combinations to determine an optimal multigene model and its core genes. *HLA-DRB1* was selected for exploratory scTenifoldKnk-based virtual knockout mainly because it was included in the optimal model and has a well-established role in RA immunogenetics; the single-cell analysis was used only to describe cellular distribution in the RA/PsA dataset. Molecular docking was then used to evaluate the possible interaction between aspartame and *HLA-DRB1*. Forty-four intersected genes linked the predicted aspartame targets with RA DEGs. The random forest plus partial least-squares generalized linear model (RF + plsRglm) identified 16 core genes. Network-level interpretation indicated that these genes were distributed across immune/antigen-processing, inflammatory-signaling, protease/extracellular-matrix-remodeling, adhesion, metabolic, and proliferation-related modules; therefore, *HLA-DRB1* was treated as a prioritized immune-module candidate rather than as the sole driver of the network. Following virtual knockout of *HLA-DRB1*, affected genes were enriched in extracellular matrix organization, extracellular structure organization, extracellular matrix, collagen trimer, extracellular matrix structural constituent, and collagen binding. Kyoto Encyclopedia of Genes and Genomes (KEGG) pathways included integrin signaling, focal adhesion, proteoglycans in cancer, cytoskeleton in muscle, and phosphoinositide 3-kinase/protein kinase B (PI3K/AKT) signaling. Molecular docking showed a minimum binding energy of −6.7 kcal/mol, which was more negative than the preset stability criterion of −5.0 kcal/mol, and the docking pose suggested contacts around ARG-146. This integrative analysis suggests a hypothesis-generating association between aspartame-related predicted targets and RA-relevant molecular networks involving *HLA-DRB1* and other core genes. The findings do not establish causality and require experimental, epidemiological, biophysical, and tissue-stratified validation before any causal or clinical inference can be made.

## 1. Introduction

Rheumatoid arthritis (RA) is a common chronic inflammatory disease that profoundly impacts patients’ quality of life through persistent joint damage and systemic complications [1]. As of 2020, approximately 17.6 million people worldwide were affected by RA, representing a 14.1% increase compared with 1990 [2]. The Global Burden of Disease (GBD) 2021 study projected that the number of RA patients will reach 31.7 million by 2050 [3]. RA undergoes a chronic inflammatory process that ultimately leads to joint deformity, damage, disability, and even death [4]. The disease typically manifests as symmetric polyarthritis, primarily affecting the small joints of the hands and feet, although large joints and other organs may also be involved [5].

Artificial sweeteners (ASWs), also known as non-nutritive sweeteners (NNSs), serve as substitutes for caloric sugars [6]. Aspartame, also referred to as L-α-aspartyl-L-phenylalanine methyl ester, is a low-calorie artificial sweetener [7]. Aspartame is widely used worldwide in more than 5000 food and beverage products, including cereals, chewing gum, yogurt, pharmaceuticals, and instant coffee [8]. It is also commonly used in the production of low-calorie beverages, including products consumed by children and pregnant women [9]. The U.S. Food and Drug Administration (FDA) has established an acceptable daily intake (ADI) for aspartame of 50 mg/kg body weight [10]. Given its widespread use, exposure assessment remains relevant, although actual intake varies substantially across products and populations. Xu et al. [11] noted that artificial sweeteners may affect bone structure and muscle metabolism by modulating gut microbiota, inducing oxidative stress, and disrupting metabolic pathways; however, direct epidemiological and experimental evidence linking aspartame to RA remains limited. Therefore, the present study focused on exploring whether predicted aspartame-related targets overlap with RA-associated molecular networks, rather than inferring that aspartame exposure causes RA.

Network toxicology, which integrates compound target prediction with disease-related gene expression profiles, serves as a powerful tool for uncovering the molecular links between pathogenic factors and diseases. This approach has shown great potential in elucidating the pathogenic mechanisms of exogenous chemicals such as sodium benzoate (SB) [12] and aflatoxin B1 (AFB1) [13]. With the advancement of bioinformatics, machine learning algorithms have been successfully applied to construct RA diagnostic models based on gene expression profiles and to identify key biomarkers, such as genes associated with cuproptosis [14] or mitophagy [15]. The application of single-cell sequencing technology has further revealed the critical role of cell heterogeneity in the pathogenesis of RA. For instance, Feng et al. [16] identified the N6-methyladenosine (m6A) methylation regulator *YTHDC1* associated with RA through single-cell analysis.

An integrative analysis that combines target prediction of exogenous compounds (such as aspartame), machine learning-based disease model construction, expression validation at the single-cell level, and functional prediction of key genes (such as virtual knockout) remains limited. In this study, we integrated network toxicology, machine learning, and single-cell analysis to investigate a potential association between aspartame-related predicted targets and RA using public data from the Gene Expression Omnibus (GEO) database. The scTenifoldKnk algorithm [17] was applied to perform exploratory virtual knockout of the prioritized core gene, simulating the impact of its functional loss on cellular regulatory networks, and molecular docking was conducted to evaluate the plausibility of a compound-protein interaction. Together, these analyses were intended to generate mechanistic hypotheses rather than to establish a causal relationship between aspartame exposure and RA.

## 2. Results

### 2.1. Data Correction and Identification of Differentially Expressed Genes

**Figure 1:**
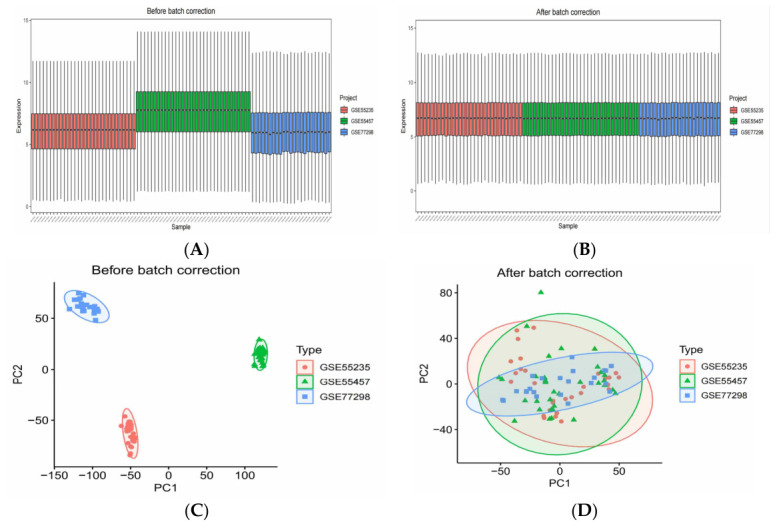

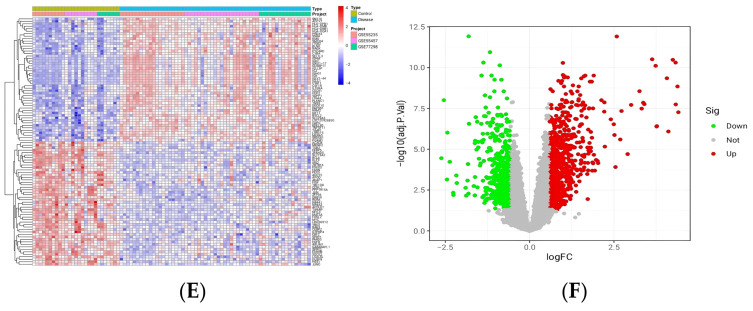
(**A**) Box plot of expression levels before batch correction; (**B**) box plot of expression levels after batch correction; (**C**) principal component analysis (PCA) plot before batch correction; (**D**) PCA plot after batch correction; (**E**) heatmap of differentially expressed genes showing the expression of the top 50 upregulated and downregulated genes across samples from the rheumatoid arthritis (RA) and control groups. Red indicates high expression and blue indicates low expression; (**F**) volcano plot displaying differentially expressed genes based on log2 fold change and significance level. Red dots represent upregulated genes, green dots represent downregulated genes, and gray dots represent genes with no significant difference.

After batch-effect correction of the three discovery datasets (GSE55235, GSE55457, and GSE77298), box plots showed that the overall expression-value distributions across samples became more concordant (Figure 1A,B). Principal component analysis (PCA) revealed that, prior to correction, samples were clearly separated by dataset origin (Figure 1C), whereas after correction, samples from different datasets were more intermixed (Figure 1D), indicating that dataset-level technical variation was reduced. However, because the integrated datasets were derived from different tissue origins, including peripheral blood and synovial tissue, tissue origin remains a major biological source of variation that cannot be fully eliminated by batch correction. Therefore, the differentially expressed genes (DEGs) identified from the integrated matrix were interpreted as mixed-tissue RA-associated transcriptional signals rather than tissue-independent RA markers. Differential expression analysis identified RA-associated DEGs, and their overall expression patterns together with the distribution of significantly altered genes were displayed through a heatmap (Figure 1E) and a volcano plot (Figure 1F).

Because tissue source was not orthogonal to dataset source and disease status in the available GEO series, the present design cannot statistically separate RA-associated transcriptional changes from tissue-composition effects. Genes preferentially expressed in blood-derived immune cells or synovial stromal compartments may therefore contribute disproportionately to the DEG set and to the subsequent machine-learning and network features. These results should consequently be read as mixed-source exploratory signals that require tissue-matched validation.

### 2.2. Single-Cell Data Clustering and Annotation

**Figure 2:**
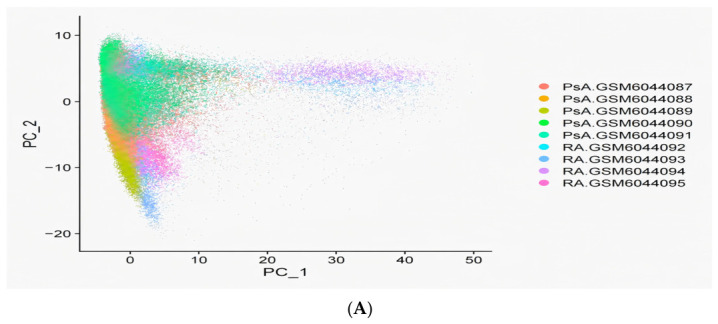

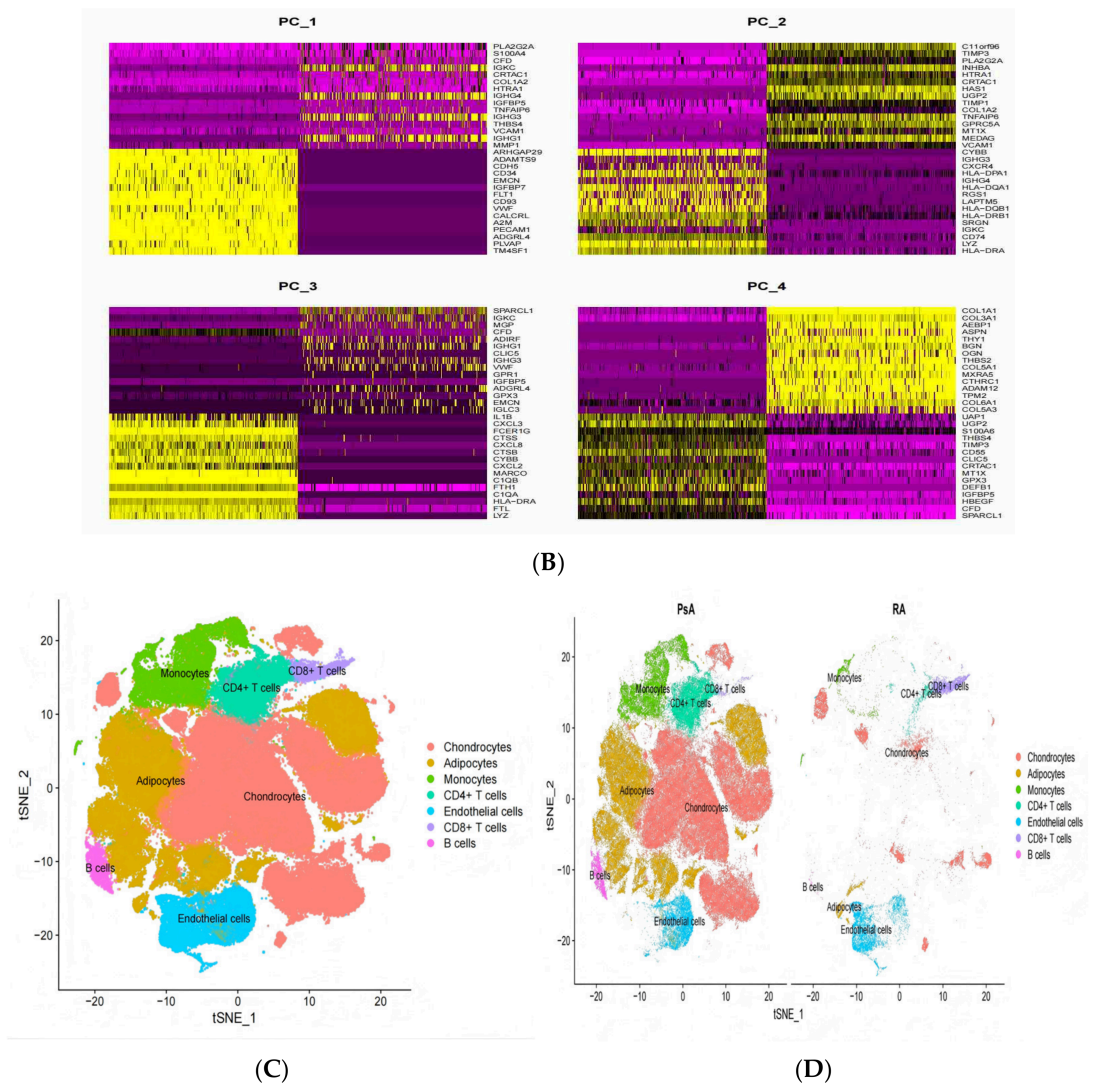
(**A**) Principal component analysis (PCA) plot of single-cell data showing distances between cells from different GSE200815 samples. (**B**) Heatmap of PCA results for single-cell data. The horizontal axis represents cell identifiers and the vertical axis represents gene names; yellow indicates high gene expression, the yellow-to-purple scale denotes relatively higher-to-lower expression in the PCA heatmap. (**C**) Cell annotation map from single-cell data, enabling identification of the cell type of each individual cell. (**D**) Cell annotation map grouped by condition (psoriatic arthritis (PsA) vs. rheumatoid arthritis (RA)), facilitating comparison of cell type composition between the psoriatic arthritis (PsA) and RA groups. In panels (**A**,**D**), different colors denote different samples or groups as shown in the legends; in panel (**C**), different colors denote annotated cell types.

A single-cell atlas was constructed by analyzing the GSE200815 dataset. PCA (Figure 2A) and a heatmap (Figure 2B) displayed the clustering of cells. Based on the expression of marker genes, the cells were primarily annotated as chondrocytes, adipocytes, monocytes, CD4+ T cells, endothelial cells, CD8+ T cells, and B cells (Figure 2C). Figure 2D revealed differences in cell composition between the psoriatic arthritis (PsA) and RA groups. Because this dataset lacks healthy controls, these differences were interpreted as cellular heterogeneity between two inflammatory arthritides rather than RA-specific changes relative to healthy individuals. Consequently, the single-cell results were used for cell-type localization and visualization only, not as independent disease-versus-control validation.

### 2.3. Prediction of Aspartame Targets and Construction of the Regulatory Network

**Figure 3:**
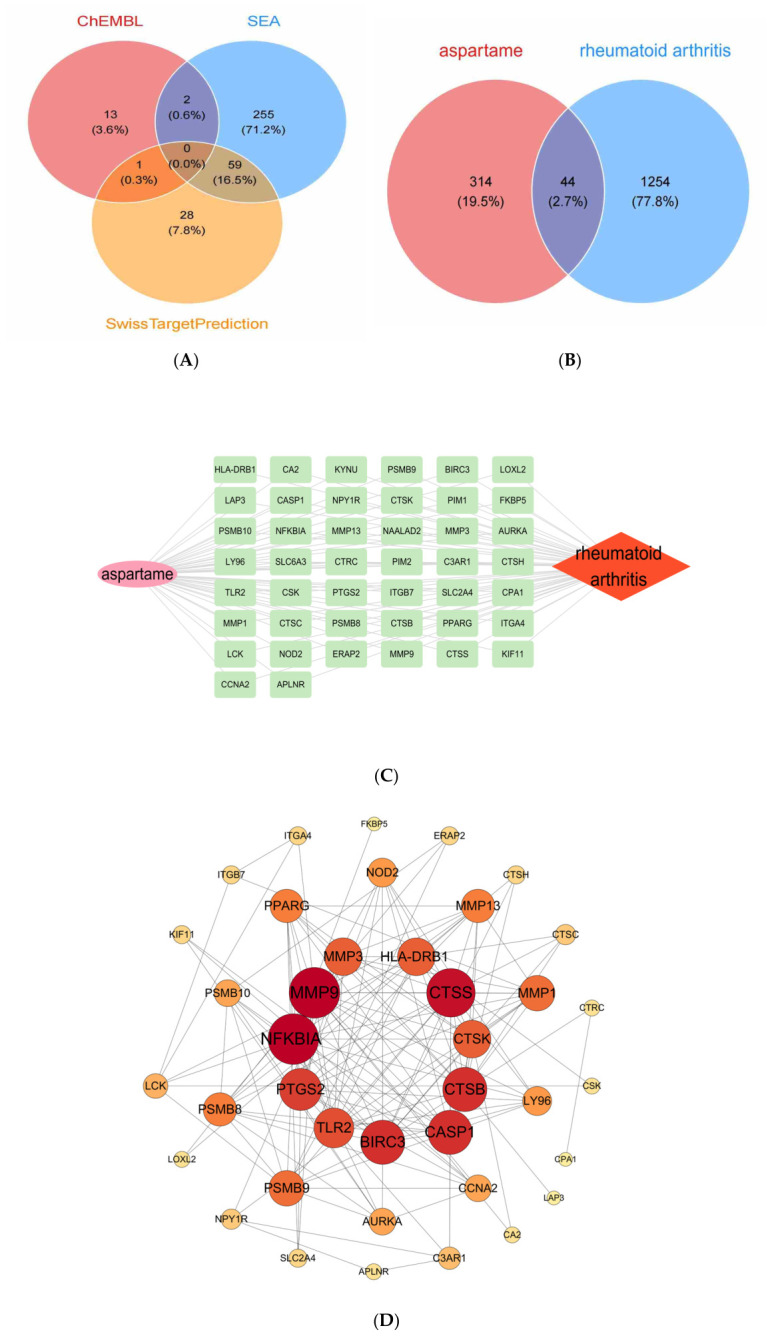
(**A**) Union of aspartame target genes predicted by the ChEMBL, SwissTargetPrediction, and SEA databases; (**B**) intersection of RA differentially expressed genes (DEGs) and potential aspartame target genes; (**C**) “Aspartame-gene-RA” regulatory network; (**D**) protein–protein interaction (PPI) network of the 44 intersected genes. Larger and darker/redder nodes represent genes with higher relative connectivity in the displayed network.

Potential aspartame targets were predicted using the ChEMBL, SwissTargetPrediction, and SEA databases. The overlap among the targets from the three databases was displayed, and a unified aspartame target set was obtained by merging all candidates (Figure 3A). This target set was then intersected with the RA-related DEGs, yielding 44 shared targets (Figure 3B), suggesting that aspartame-related predicted targets overlap with a subset of RA-associated transcriptional alterations. Based on these shared targets, a compound-gene-disease regulatory network was constructed (Figure 3C), which visually depicts the associations among aspartame, the target genes, and RA.

To avoid reducing this 44-gene backbone to a single-gene explanation, the intersected genes were further interpreted at the network/module level. The 44 genes comprised several biologically coherent groups, including immune and antigen-processing genes (*HLA-DRB1*, *PSMB8*, *PSMB9*, *PSMB10*, and *ERAP2*), innate inflammatory-signaling genes (*TLR2*, *LY96*, *NOD2*, *CASP1*, *NFKBIA*, *BIRC3*, and *PTGS2*), protease and extracellular matrix (ECM) remodeling genes (*MMP1*, *MMP3*, *MMP9*, *MMP13*, *CTSB*, *CTSC*, *CTSH*, *CTSK*, and *CTSS*), and adhesion-, metabolic-, and proliferation-related genes (*ITGA4*, *ITGB7*, *LCK*, *CSK*, *CCNA2*, *KIF11*, *PPARG*, and *SLC2A4*). This module-level pattern indicates that any biological effect, if present, is more plausibly distributed across a gene network than concentrated on *HLA-DRB1* alone. Accordingly, *HLA-DRB1* was regarded as a prioritized immune-module candidate for downstream hypothesis generation, not as a proven network hub or exclusive mediator. To further support this interpretation, the 44 intersected genes were imported into STRING and visualized as a protein-protein interaction (PPI) network in Cytoscape (Figure 3D). Topology was assessed descriptively by relative connectivity and bridge-like position in the displayed network. Inflammatory nodes such as *CASP1*, *NFKBIA*, *TLR2*, *BIRC3*, and *PTGS2* and ECM/protease-related nodes such as *MMP3*, *MMP9*, *CTSB*, *CTSK*, and *CTSS* occupied relatively connected or bridging positions, whereas *HLA-DRB1* was embedded within the antigen-processing/immune region but did not appear to be the sole dominant hub. The aspartame-overlap genes were therefore distributed across several related modules rather than clustering exclusively around *HLA-DRB1*. This topology supports interpreting the predicted aspartame-RA association as a distributed gene-network hypothesis rather than a single HLA-DRB1-dependent mechanism.

### 2.4. Identification of Core Genes by Machine Learning Models

**Figure 4:**
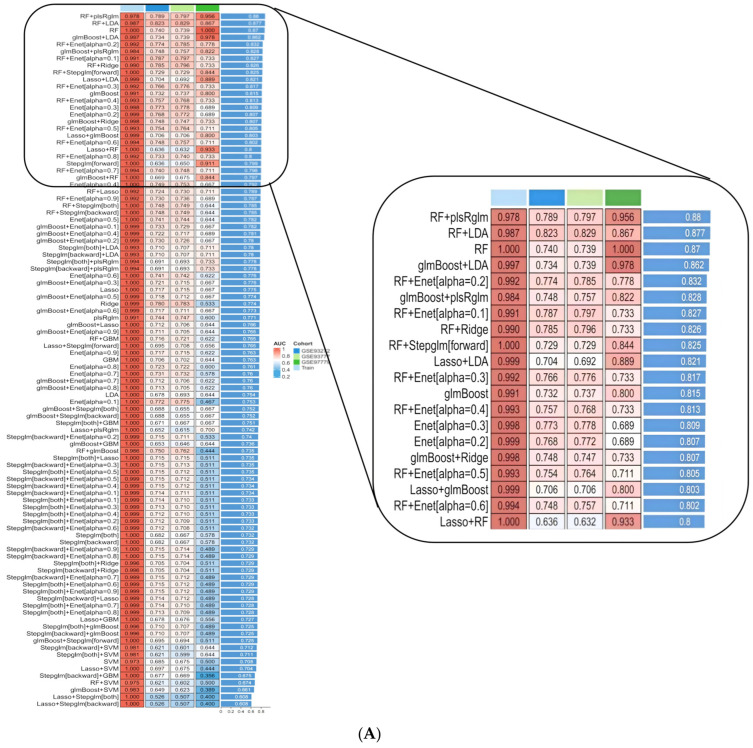

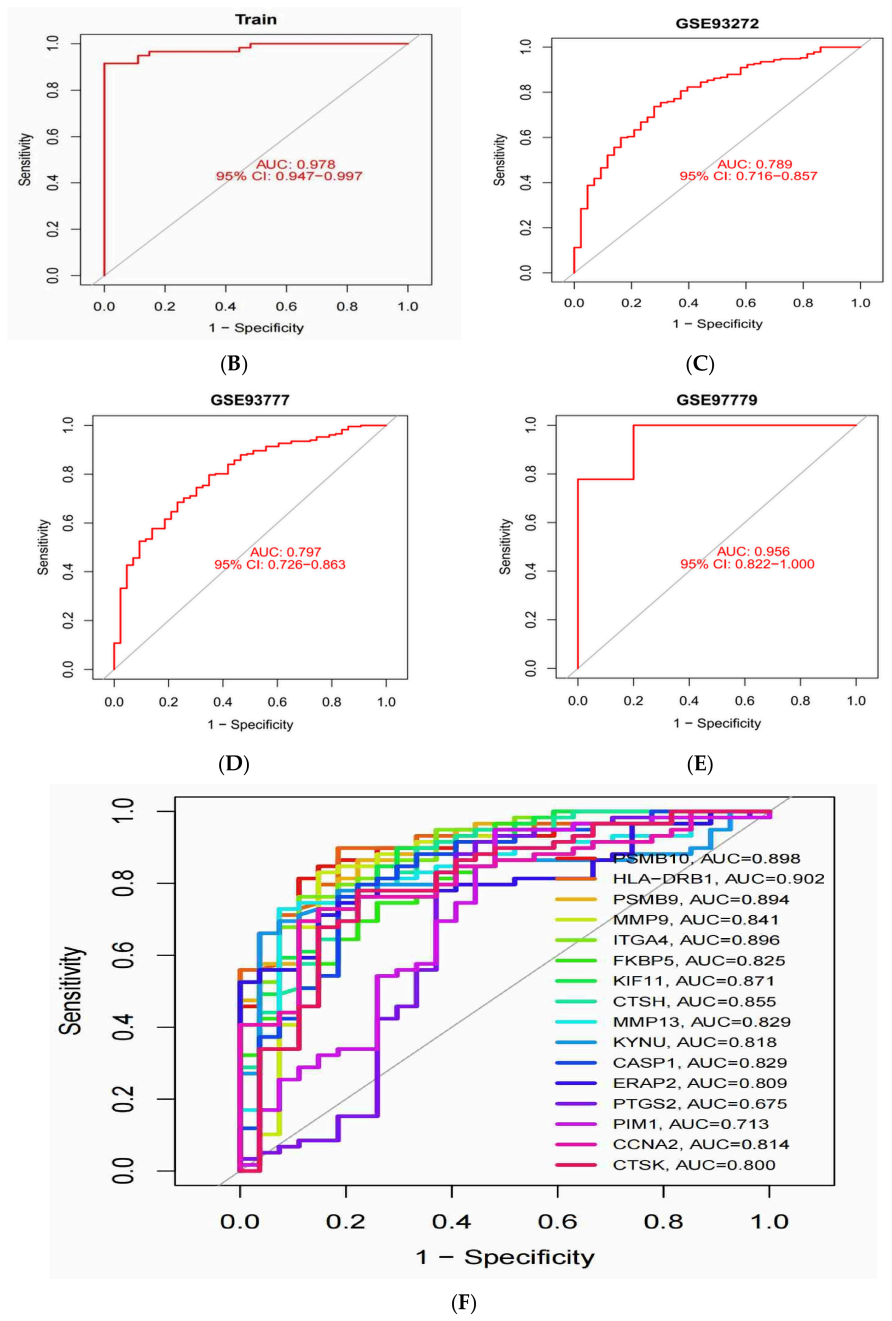
(**A**) Classification performance heatmap of multiple model combinations: the vertical axis represents combinations of machine learning models, the horizontal axis represents data sources, and the values represent the area under the receiver operating characteristic curve (AUC); (**B**) receiver operating characteristic (ROC) curve for the training set; (**C**–**E**) receiver operating characteristic (ROC) curves for the validation sets; (**F**) receiver operating characteristic (ROC) curves of the core genes.

Using the 44 intersected genes as features, the optimal diagnostic model was identified from 113 machine-learning algorithm combinations (Figure 4A). To reduce direct information leakage, DEG screening, target intersection, feature ranking, model construction, internal resampling, hyperparameter tuning, and model selection were performed within the discovery/training datasets, and the external validation datasets were used only after the model and core-gene set had been fixed. Internal validation was implemented as a cross-validation-based resampling procedure within the discovery matrix; in each resampling cycle, feature ranking, model fitting, and parameter selection were refitted in the internal training subset, whereas the held-out subset was used only to estimate discrimination. Algorithm-specific tuning, when required, was based on cross-validated area under the receiver operating characteristic curve (AUC) within the discovery matrix and was completed before any external validation cohort was evaluated. AUC confidence intervals were calculated with the pROC workflow, using DeLong-type intervals where applicable. The random forest plus partial least-squares generalized linear model (RF + plsRglm) demonstrated the best overall performance during training and external validation. On the training set, the model achieved an AUC of 0.978 (Figure 4B), and on the independent validation sets GSE93272, GSE93777, and GSE97779, it yielded AUCs of 0.789, 0.797, and 0.956, respectively (Figure 4C–E). The reported receiver operating characteristic (ROC) curves included 95% confidence intervals where available. Ultimately, 16 core genes with the greatest contributions to the model were identified: *PSMB10*, *HLA-DRB1*, *PSMB9*, *MMP9*, *ITGA4*, *FKBP5*, *KIF11*, *CTSH*, *MMP13*, *KYNU*, *CASP1*, *ERAP2*, *PTGS2*, *PIM1*, *CCNA2*, and *CTSK*. In general, an AUC ≥ 0.6 is considered moderate, and an AUC ≥ 0.7 is considered good [18]. The individual ROC curves of these genes showed diagnostic value for most genes, whereas *PTGS2* showed poor individual discrimination (AUC = 0.500; Figure 4F). Therefore, *PTGS2* was excluded from the subsequent single-cell visualization step, although it remained part of the multigene model output. Because the AUC in GSE97779 was relatively high (0.956), this result was interpreted cautiously as a cohort-specific external validation result rather than as proof of generalizable diagnostic performance. GSE97779 was not used for DEG identification, feature selection, model training, or model selection; nevertheless, its cohort composition, sample size, and possible residual platform effects may still influence the estimate. Therefore, model performance was considered across all validation cohorts, including the lower AUCs observed in GSE93272 and GSE93777, rather than being inferred from GSE97779 alone. Calibration metrics, including calibration slope, calibration intercept, and Brier score, were not reported because the model was designed for network-guided feature prioritization rather than clinical risk prediction. Accordingly, the model was interpreted as an exploratory multigene prioritization tool rather than as a calibrated clinical diagnostic model.

The internal validation was intended to rank candidate algorithms and control direct leakage, not to establish a deployable clinical risk model. Within each internal split, feature handling and model-specific tuning were repeated using only the corresponding internal training subset, and the held-out subset was used only for discrimination assessment. The external cohorts were examined only after the RF + plsRglm workflow and the 16-gene panel had been fixed. Because the candidate algorithms generated heterogeneous model scores rather than a prespecified probability scale, calibration indices were not used to support clinical calibration.

### 2.5. Expression of Core Genes in the Single-Cell Atlas

**Figure 5:**
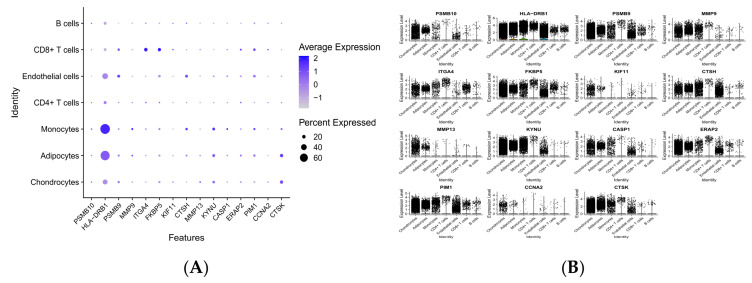
(**A**) Dot plot of core genes from the model visualized on single-cell data: the horizontal axis represents the core genes used in model construction, the vertical axis represents cell types, dot size indicates the percentage of expressing cells, and color intensity represents the average expression level (darker color indicates higher expression); (**B**) violin plot of core genes from the model visualized on single-cell data: the horizontal axis represents cell types, and the vertical axis represents gene expression levels.

To elucidate the cellular distribution of the core genes, the 15 core genes excluding *PTGS2* were mapped onto the RA/PsA single-cell dataset. *PTGS2* was excluded from Figure 5 because its individual ROC performance was poor (AUC = 0.500), as described above. Visualization using dot plots (Figure 5A) and violin plots (Figure 5B) revealed that these genes exhibited expression patterns across distinct cell types in the RA/PsA dataset. Among them, *HLA-DRB1* showed high average expression levels and a broad percentage of expressing cells across multiple cell types, with prominent expression in monocytes as well as in stromal cells such as adipocytes and chondrocytes. Because GSE200815 does not include healthy controls, these single-cell findings were not interpreted as RA-specific disease-versus-healthy-control evidence. *HLA-DRB1* was selected for further exploratory virtual gene knockout analysis primarily because of its inclusion in the multigene model and its established role in RA immunogenetics; the single-cell analysis provided only cellular-context information within the RA/PsA dataset.

### 2.6. Functional Prediction of HLA-DRB1 Virtual Knockout

**Figure 6:**
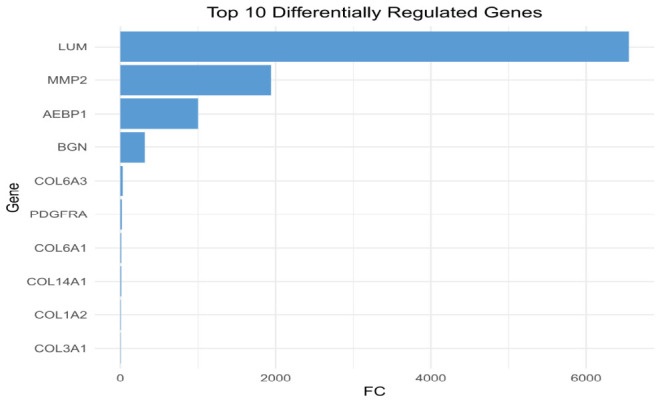
Bar plot of the top 10 most significantly altered genes following virtual knockout.

After virtual knockout of *HLA-DRB1* using the scTenifoldKnk tool, a series of genes predicted to be differentially perturbed after *HLA-DRB1* deletion were identified. A bar plot (Figure 6) displays the top 10 most strongly predicted perturbations, among which *LUM*, *MMP2*, *AEBP1*, *BGN*, *COL6A3*, and *PDGFRA* exhibited the most prominent changes. As shown in Table 1, *LUM* and *MMP2* displayed the most significant differential perturbation (adjusted *p* values reported as 0 by the software output), with fold change (FC) values as high as 6549.66 and 1941.39, respectively. These values should be understood as model-derived perturbation scores rather than experimentally measured changes. Genes such as *AEBP1*, *BGN*, *COL6A3*, and *PDGFRA* also demonstrated significant predicted perturbation (adjusted *p* value < 0.05).

### 2.7. Functional Enrichment Analysis

**Figure 7:**
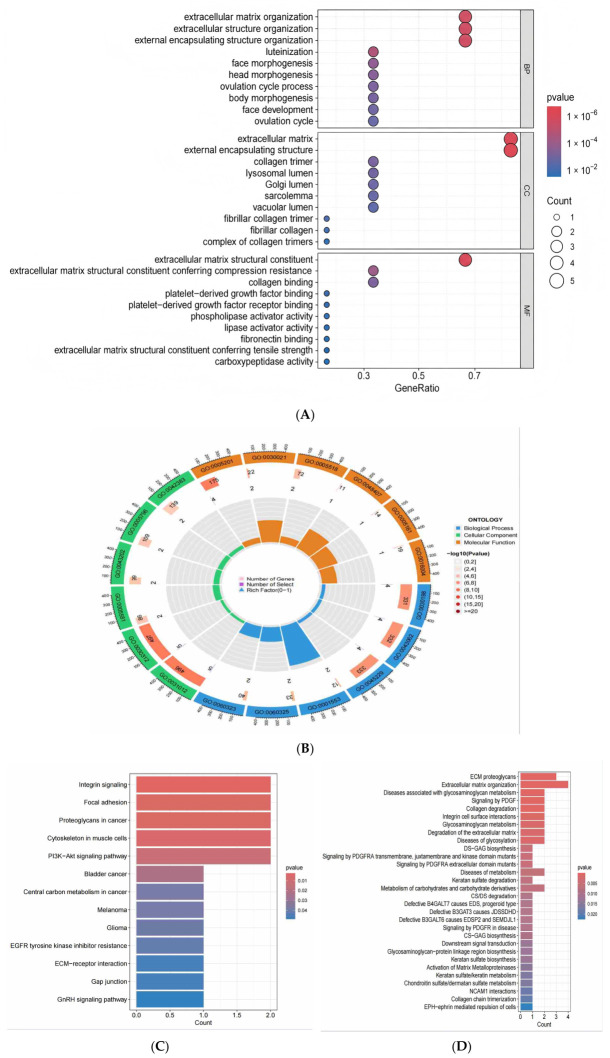
(**A**) Gene Ontology (GO) bubble plot: the horizontal axis represents the gene ratio, and the vertical axis represents pathway names. The size of the circles indicates the number of genes enriched in each pathway, and the color represents the enrichment significance—a redder color indicates more significant enrichment of differentially expressed genes in that pathway. (**B**) GO circle plot: the outermost ring represents the total number of genes (expressed as powers of 10); the first inner ring represents pathway IDs; the second inner ring indicates the number of genes contained in each term, with darker colors reflecting more significant enrichment; the third ring shows the number of differentially expressed genes enriched in each GO term; and the fourth ring represents the ratio of the gene count in the third ring to that in the second ring. (**C**) Kyoto Encyclopedia of Genes and Genomes (KEGG) bar plot: the horizontal axis represents the number of genes enriched in each pathway, and the vertical axis represents pathway names. The color of the bars indicates the enrichment significance—a redder color indicates more significant enrichment of differentially expressed genes in that pathway. (**D**) Reactome bar plot: the horizontal axis represents the number of genes enriched in each pathway, and the vertical axis represents pathway names. The color of the bars indicates the enrichment significance—a redder color indicates more significant enrichment of differentially expressed genes in that pathway.

Gene Ontology (GO) enrichment analysis (Figure 7A) showed that genes predicted to be affected by *HLA-DRB1* virtual deletion were enriched in biological processes and cellular components such as “extracellular matrix organization,” “extracellular structure organization,” “extracellular matrix,” and “collagen trimer,” suggesting that the computational perturbation may be linked to extracellular matrix (ECM)-related processes in the RA/PsA single-cell context. For molecular functions, the affected genes were mainly enriched in terms including “extracellular matrix structural constituent” and “collagen binding.” Kyoto Encyclopedia of Genes and Genomes (KEGG) pathway enrichment analysis (Figure 7C) further showed enrichment in pathways previously reported in RA-related biology, including integrin signaling, focal adhesion, proteoglycans in cancer, cytoskeleton in muscle, and the phosphoinositide 3-kinase/protein kinase B (PI3K/AKT) signaling pathway. Reactome enrichment analysis (Figure 7D) identified enrichment mainly in pathways such as “extracellular matrix degradation,” “collagen degradation,” and “glycosaminoglycan metabolism.” These results should be interpreted as computational pathway hypotheses rather than evidence that aspartame directly disrupts immune or extracellular matrix homeostasis.

### 2.8. Molecular Docking Analysis of a Possible Aspartame-HLA-DRB1 Interaction

**Figure 8:**
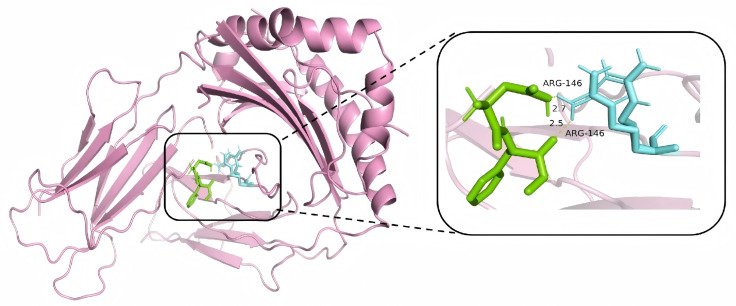
Molecular docking diagram of aspartame binding to HLA-DRB1. Aspartame is shown as the ligand, HLA-DRB1 as the receptor protein, and atom/residue colors follow the molecular visualization scheme, with ligand atoms colored by element (e.g., carbon, oxygen, and nitrogen in distinct colors); dashed lines indicate predicted contacts.

To further evaluate a possible interaction between aspartame and the core target HLA-DRB1, molecular docking simulation was performed. The docking results showed that the binding energy between aspartame and HLA-DRB1 protein was −6.7 kcal/mol (Figure 8), which was more negative than the preset docking stability criterion of −5.0 kcal/mol. The docking pose suggested contacts around ARG-146, with interaction distances of approximately 2.5 and 2.7 Å in the displayed conformation. These results support the structural plausibility of ligand binding; however, docking alone cannot determine whether aspartame alters antigen presentation, peptide-groove occupancy, HLA-DRB1 surface expression, or downstream immune signaling. Therefore, this docking analysis was used only as a structural screening step and not as biological validation of a functional interaction.

## 3. Discussion

By integrating multiple bioinformatics approaches, including network toxicology, machine learning, single-cell transcriptomic analysis, and virtual gene knockout, this study identified a potential molecular association between aspartame-related predicted targets and RA-relevant gene networks. A diagnostic model with high performance for RA was constructed, and 16 core genes were identified. *HLA-DRB1* was prioritized for downstream exploration because it appeared in the optimal multigene model and is biologically central to RA immunogenetics; the RA/PsA single-cell atlas was used only to describe the cellular distribution of this candidate in an inflammatory-arthritis context. However, the present findings should be interpreted as hypothesis-generating evidence rather than proof that aspartame causes RA or specifically acts through *HLA-DRB1* in vivo.

Among the 16 core genes identified in this study, several have been previously reported to be associated with RA. For instance, *HLA-DRB1* represents the strongest genetic susceptibility gene for RA; the protein it encodes can activate autoreactive T cells by presenting citrullinated antigens, which is considered an important step in RA pathogenesis [19]. *MMP9* is a key matrix metalloproteinase involved in cartilage and bone destruction in RA [20]. *ITGA4*, as an important adhesion molecule, mediates the migration of lymphocytes to the inflamed synovium [21]. These findings support the biological plausibility of the multigene model, but they also indicate that the recovery of *HLA-DRB1* is partly expected from RA biology and should not be overinterpreted as independent evidence of aspartame-specific targeting.

RA is a complex polygenic and network-driven disease, and the 16 core genes should be viewed collectively. The network-level pattern of the 44 intersected genes suggests distributed involvement of antigen processing, innate immune signaling, protease activity, extracellular matrix remodeling, adhesion, metabolism, and cell proliferation. Within this framework, *HLA-DRB1* may act as an immune-contextual anchor, whereas genes such as *PSMB8*, *PSMB9*, *PSMB10*, *ERAP2*, *CASP1*, *NFKBIA*, *MMP9*, *MMP13*, *ITGA4*, and *CTSK* may contribute complementary information to the model. Therefore, the biological effects suggested by this analysis are more consistent with a network-level hypothesis than with a single-gene narrative.

This prioritization carries a potential circularity because *HLA-DRB1* is already a well-established RA susceptibility gene. We therefore interpret its selection cautiously. *HLA-DRB1* was not considered important simply because it is known in RA; rather, it was selected after two main layers of evidence converged: overlap between predicted aspartame targets and RA-associated DEGs and inclusion in the optimal multigene model. The single-cell dataset provided cellular-distribution information only and did not constitute RA-versus-healthy-control validation. Even so, these layers do not demonstrate causality. They indicate that *HLA-DRB1* is a reasonable candidate for mechanistic follow-up within a larger network, not that it is the exclusive or experimentally validated target of aspartame.

Virtual knockout of *HLA-DRB1* using scTenifoldKnk revealed that loss of *HLA-DRB1* was predicted to perturb genes such as *LUM* and *MMP2*. Previous studies have shown that serum LUM is expressed in RA patients and is significantly elevated compared with healthy controls, suggesting a potential biological association between *LUM* and RA disease activity indicators. *LUM* levels were also positively correlated with serum IgA and rheumatoid factor levels, indicating that *LUM* may reflect the immune activity of RA [22]. Immunostaining for *MMP2* has been detected in both synoviocytes and CD34+ vascular endothelial cells within the synovial tissue of RA patients, suggesting that *MMP2* is involved in RA progression and plays an important role in the formation and invasion of rheumatoid synovium [23]. Because scTenifoldKnk is a computational perturbation method, these results should be interpreted as predicted network responses that require experimental confirmation.

Based on enrichment analysis after virtual knockout of *HLA-DRB1*, the affected genes were enriched in biological processes and cellular components such as “extracellular matrix organization,” “extracellular structure organization,” “extracellular matrix,” and “collagen trimer,” suggesting that the predicted perturbation is connected to extracellular matrix (ECM) homeostasis. These genes were also implicated in molecular functions including “extracellular matrix structural constituent” and “collagen binding.” KEGG enrichment analysis further revealed pathways associated with RA pathogenesis, including integrin signaling, focal adhesion, proteoglycans in cancer, cytoskeleton in muscle, and the PI3K/AKT signaling pathway. Lowin et al. [24] indicated that RA is at least partially an integrin-driven disease. Other studies have linked focal adhesion [25], cytoskeletal autoantigens [26], and PI3K/AKT signaling [27,28,29] to RA-related inflammation, synovial activation, and fibroblast-like synoviocyte behavior. Reactome enrichment analysis mainly highlighted “extracellular matrix degradation,” “collagen degradation,” and “glycosaminoglycan metabolism.” These findings are consistent with the known importance of ECM remodeling in RA [30,31], collagen degradation under inflammatory conditions [32], and altered hyaluronic-acid-dependent joint lubrication [33]. Nevertheless, these pathway results derive from a virtual knockout model and should be viewed as mechanistic hypotheses connecting immune regulation and matrix remodeling rather than direct experimental evidence of aspartame-induced RA.

In molecular docking, a binding energy ≤ −5.0 kcal/mol is generally considered to indicate acceptable binding stability between the receptor and ligand [34]. In this study, the minimum binding energy was −6.7 kcal/mol, and the displayed docking pose indicated potential contacts near ARG-146. This result is compatible with moderate binding stability, but it is insufficient by itself to establish a biologically meaningful interaction. In particular, whether this pose affects antigen presentation, peptide-groove occupancy, HLA-DRB1 surface expression, or immune-cell activation remains unknown. Future validation should include biophysical assays such as surface plasmon resonance or isothermal titration calorimetry, followed by functional assays in antigen-presenting cells.

The machine-learning results also require cautious interpretation. The AUC of 0.956 in GSE97779 may reflect true cohort separability, but it may also be influenced by sample size, cohort composition, platform differences, or residual batch effects. In addition, because candidate features were restricted to RA-associated DEGs before model construction, the workflow may introduce feature-selection circularity and inflate apparent discrimination. For this reason, the multigene model is presented as a network-guided feature-prioritization result and not as a clinically validated diagnostic classifier.

Alternative interpretations should also be considered. The observed bioinformatic associations may reflect general inflammatory status, disease-associated changes in diet or medication use, altered metabolism of sweeteners under inflammatory conditions, or shared immune pathways between RA and other inflammatory arthritides rather than a direct effect of aspartame on RA onset. Therefore, prospective exposure data and experimental models are necessary to evaluate temporal directionality and causality.

## 4. Methods

### 4.1. Data Acquisition and Preprocessing

No experimental chemicals, reagents, devices, instruments, commercial cell lines, samples, or materials were used in this study; all analyses were based on publicly available datasets, public databases, and computational software. Three RA transcriptomic datasets derived from peripheral blood or synovial tissue (GSE55235, GSE55457, and GSE77298) were obtained from the Gene Expression Omnibus (GEO) database (https://www.ncbi.nlm.nih.gov/geo/ (accessed on 18 January 2026)) and used only as discovery/training datasets. Normalized GEO expression matrices were used when available. Probe identifiers were mapped to official gene symbols according to the corresponding platform annotation files; probes mapping to the same gene symbol were collapsed by mean expression, and genes shared by the discovery datasets were retained before merging. Expression values were inspected for scale consistency, and log2 transformation was applied when required according to the expression-value distribution. Batch-effect correction in the discovery matrix was performed using the removeBatchEffect function from the limma package in R software (R version 4.5.2; limma documentation: https://bioconductor.org/packages/limma/ (accessed on 2 February 2026)), with dataset origin specified as the batch variable. Tissue origin was not treated as a purely technical batch variable because it represents a major biological source of variation. Because the available discovery datasets did not provide a balanced design in which tissue origin could be fully separated from dataset source and disease status, a formal tissue-stratified sensitivity analysis was not performed. Therefore, downstream DEGs and model features were interpreted as mixed-tissue RA-associated signals. Datasets GSE93272, GSE93777, and GSE97779 were downloaded and processed separately as independent validation sets; they were not used for DEG screening, feature selection, model tuning, or model selection. The single-cell RNA sequencing dataset GSE200815 was utilized for subsequent cell clustering and annotation; this dataset includes RA and PsA samples, and therefore its group comparison was defined as RA-vs-PsA.

In the available discovery matrices, tissue category, dataset identity, and disease/control composition were partially confounded. Adding tissue origin as a conventional adjustment covariate would therefore not provide an independent estimate of tissue effect and could remove genuine disease-related variation. For the same reason, a formal tissue-stratified sensitivity analysis was not statistically reliable. Batch correction was accordingly limited to dataset-level technical variation, while tissue origin was retained as an explicit biological constraint on interpretation.

### 4.2. Identification of Differentially Expressed Genes

The combined batch-corrected dataset (GSE55235, GSE55457, and GSE77298) was analyzed using the limma package in R software (R version 4.5.2; limma documentation: https://bioconductor.org/packages/limma/ (accessed on 2 February 2026)) to identify differentially expressed genes (DEGs) between the RA group and healthy controls. Genes were considered significantly differentially expressed if they met the thresholds of adjusted p value < 0.05 and |log2FC| > 0.585 (corresponding to a 1.5-fold change). The results of differential expression analysis were visualized using volcano plots and heatmaps. DEG screening was restricted to the discovery/training datasets. The independent validation cohorts were not included in differential expression analysis, target intersection, or feature selection, thereby reducing direct information leakage from validation data into model construction.

### 4.3. Single-Cell Data Clustering and Annotation

Quality control, normalization, dimensionality reduction by principal component analysis (PCA), and clustering analysis were performed on the GSE200815 dataset using the Seurat package in R software (R version 4.5.2; Seurat documentation: https://satijalab.org/seurat/ (accessed on 2 February 2026)). Cells with mitochondrial gene proportion > 10% or library size < 1000 were removed before downstream analysis, consistent with the thresholds used in the subsequent scTenifoldKnk preprocessing. The remaining cells were normalized using the LogNormalize workflow, variable features were selected, expression values were scaled, and PCA was performed for dimensionality reduction. Cell clusters were annotated using the SingleR package (https://bioconductor.org/packages/SingleR/ (accessed on 2 February 2026)) and visualized using the t-distributed stochastic neighbor embedding (t-SNE) algorithm implemented in Rtsne (https://cran.r-project.org/package=Rtsne (accessed on 2 February 2026)). Differences in cell type composition were compared between the RA and PsA groups.

### 4.4. Prediction of Potential Aspartame Targets and Network Construction

Potential targets of aspartame were predicted using three publicly available databases. (1) ChEMBL database (https://www.ebi.ac.uk/chembl/ (accessed on 25 March 2026)): “aspartame” was used as a keyword to retrieve its known or predicted target proteins. (2) SwissTargetPrediction database (http://www.swisstargetprediction.ch/ (accessed on 25 March 2026)): the Simplified Molecular Input Line Entry System (SMILES) string of aspartame obtained from PubChem (https://pubchem.ncbi.nlm.nih.gov/ (accessed on 25 March 2026)) was submitted to predict targets, and those with a probability > 0.05 were retained. (3) Similarity Ensemble Approach (SEA) database (https://sea.bkslab.org/ (accessed on 25 March 2026)): the same SMILES string was queried, and targets belonging to *Homo sapiens* were screened. The predicted targets from the three databases were merged and deduplicated to generate a comprehensive set of potential aspartame targets. This target set was then intersected with the RA DEGs obtained in Section 4.2 to identify the potential targets through which aspartame may be associated with RA-related transcriptional changes. Finally, an “aspartame-intersected targets-RA” regulatory network was constructed using Cytoscape 3.10.3. To reduce single-gene overinterpretation, the 44 intersected genes were additionally interpreted at the module level according to their known biological functions, including antigen processing, inflammatory signaling, ECM/protease activity, adhesion, metabolism, and proliferation. The 44 intersected genes were submitted to STRING (https://string-db.org/ (accessed on 25 March 2026)) to construct a protein-protein interaction (PPI) network, and the resulting network was visualized in Cytoscape. Relative connectivity and bridge-like positions were inspected with Cytoscape NetworkAnalyzer-style topology assessment, including degree- and betweenness-related interpretation. Node size and color were used to indicate relative connectivity in the displayed network. This analysis was used to describe whether *HLA-DRB1* was embedded in a broader gene network and whether the intersected genes were distributed across multiple modules, rather than to infer direct experimental protein-protein binding or causality.

### 4.5. Construction of Machine Learning Diagnostic Models and Identification of Core Genes

The intersected genes between DEGs and aspartame targets were used as candidate features, with RA status (healthy control/disease) as the label, to construct machine-learning diagnostic models on the discovery/training set (GSE55235, GSE55457, and GSE77298). Twelve feature-selection/modeling algorithms (including Lasso, Ridge, Enet, Stepglm, support vector machine (SVM), glmBoost, linear discriminant analysis (LDA), plsRglm, random forest (RF), gradient boosting machine (GBM), and NaiveBayes) and their combinations were applied, yielding 113 algorithm combinations to identify the model with the best diagnostic performance and its optimal core gene set. Internal validation was performed entirely within the discovery/training matrix using a cross-validation-based resampling workflow under a fixed random seed. During each resampling cycle, candidate-feature ranking, model fitting, and parameter selection were repeated in the internal training subset, and the corresponding held-out subset was used only for estimating classification performance. Penalized regression models were tuned by selecting the regularization parameter through the cross-validation routine of the corresponding R implementation. For tree-, boosting-, support-vector-, and partial-least-squares-based learners, candidate hyperparameters were optimized within the same training resampling workflow according to cross-validated area under the receiver operating characteristic curve (AUC) when the algorithm required explicit tuning; algorithms without a required tuning step were evaluated with the package default settings but remained within the same resampling framework. Model construction, feature ranking, internal resampling, hyperparameter tuning, and model selection were performed only within the discovery/training matrix. For algorithms requiring tuning, hyperparameters were optimized within the training workflow rather than on the external validation cohorts. The independent validation datasets GSE93272, GSE93777, and GSE97779 were not used during DEG screening, target intersection, feature selection, model training, parameter selection, or algorithm selection. They were used only after the model was locked to estimate out-of-sample discrimination, and ROC curves were generated separately for each validation cohort. AUC values and 95% confidence intervals were reported for discrimination. The 95% confidence intervals for ROC analyses were estimated using the pROC package, with DeLong-type intervals applied when supported by the cohort structure. Calibration slope, calibration intercept, and Brier score were not calculated because the present model was developed for network-guided feature prioritization and did not prespecify individualized risk probabilities or a clinical decision threshold. Furthermore, individual ROC curves for the core genes in the optimal model were plotted to assess their single-gene diagnostic performance. Because the candidate feature space was prefiltered by RA differential expression and predicted aspartame targets, this workflow should be regarded as network-guided feature prioritization rather than an unbiased clinical diagnostic-model development pipeline. The modelling workflow was implemented in R software (version 4.5.2) with package-specific implementations; receiver operating characteristic (ROC) analysis used pROC (https://cran.r-project.org/package=pROC (accessed on 26 March 2026)).

To clarify the leakage-control procedure, the internal workflow was treated as resampling-based model development rather than post hoc validation. For each internal split, feature ranking or selection was recalculated from the internal training subset, model-specific parameters were chosen within that subset when tuning was required, and the fitted model was then applied to the corresponding held-out subset. Penalized models used the cross-validation routine of their R implementation to select the regularization parameter; RF, GBM, SVM, and plsRglm-based learners were tuned within the same internal training workflow when explicit tuning was required, whereas algorithms without a required tuning step were kept at package defaults. No tuning decision was made using GSE93272, GSE93777, or GSE97779. These cohorts were reserved for final locked-model ROC assessment only. Because the analysis did not prespecify a clinical decision threshold or a calibrated risk scale, calibration slope, calibration intercept, and Brier score were not used to claim clinical diagnostic calibration.

### 4.6. Visualization of Core Genes in Single-Cell Data

The 16 core genes identified by machine learning (*PSMB10*, *HLA-DRB1*, *PSMB9*, *MMP9*, *ITGA4*, *FKBP5*, *KIF11*, *CTSH*, *MMP13*, *KYNU*, *CASP1*, *ERAP2*, *PTGS2*, *PIM1*, *CCNA2*, and *CTSK*) were mapped onto the single-cell data. Dot plots and violin plots were used to display the average expression levels and the percentage of expressing cells for these genes across different cell types. Before visualization, individual ROC performance was reviewed. *PTGS2* was excluded from Figure 5 because its individual ROC AUC was 0.500, which indicated poor single-gene discrimination; this exclusion affected only the visualization and did not alter the multigene model.

### 4.7. Virtual Gene Knockout Based on scTenifoldKnk

Based on the single-cell expression profiles, *HLA-DRB1* was selected as the exploratory virtual-knockout target because it was included in the optimal multigene model and is biologically relevant to immune responses and RA immunogenetics. The RA/PsA single-cell dataset was used to confirm that *HLA-DRB1* was detectable across annotated cell types and to provide cellular context, but it was not used as disease-versus-healthy-control evidence for *HLA-DRB1* prioritization. The scTenifoldKnk tool [17] (https://github.com/cailab-tamu/scTenifoldKnk (accessed on 27 March 2026)) was applied to extract the top 10,000 highly variable genes for simulated gene knockout analysis, and the top 20,000 cells with the highest mean expression were selected for subsequent analysis. Parameters were set as follows: mitochondrial content threshold = 0.1, library size threshold = 1000, number of subnetworks = 10, and 500 cells randomly sampled from each subnetwork; default settings were otherwise used unless specified. A gene regulatory network was constructed from the wild-type single-cell data, and *HLA-DRB1* was virtually deleted from this network. Manifold alignment was performed to compare the network before and after deletion, and genes that were significantly perturbed were identified. These outputs were interpreted as predicted network perturbations and not as experimentally verified downstream targets of *HLA-DRB1*.

### 4.8. Functional Enrichment Analysis

The DEGs identified after virtual knockout of *HLA-DRB1* were subjected to functional enrichment analysis. Gene Ontology (GO) enrichment analysis (including biological process, cellular component, and molecular function) and Kyoto Encyclopedia of Genes and Genomes (KEGG) pathway enrichment analysis were performed using the clusterProfiler package in R software (R version 4.5.2; clusterProfiler documentation: https://bioconductor.org/packages/clusterProfiler/ (accessed on 28 March 2026)). To further elucidate the relevant biological pathways, Reactome pathway enrichment analysis was also conducted using ReactomePA-related resources (https://bioconductor.org/packages/ReactomePA/ (accessed on 28 March 2026)).

### 4.9. Molecular Docking

The three-dimensional structure of the HLA-DRB1 protein was retrieved from the Protein Data Bank (PDB) (https://www.rcsb.org/ (accessed on 29 March 2026)). The small-molecule structure of aspartame was downloaded from the PubChem database (https://pubchem.ncbi.nlm.nih.gov/ (accessed on 29 March 2026)). Molecular docking was performed using AutoDock Tools (https://autodocksuite.scripps.edu/adt/ (accessed on 29 March 2026)), with the docking box centered on the selected binding pocket. Semi-flexible docking was carried out via AutoDock Vina (https://vina.scripps.edu/ (accessed on 29 March 2026)) to calculate the binding energy, and the conformation with the lowest binding energy was selected for visualization analysis. A binding energy threshold of ≤−5.0 kcal/mol was used as the criterion for acceptable docking stability. Residues and distances shown in the best pose were inspected to describe the binding site. However, docking was treated only as structural screening; no effect on antigen presentation, peptide-groove occupancy, HLA-DRB1 surface expression, or immune signaling was inferred from docking alone.

## 5. Limitations

This study has several limitations. First, all analyses were based on transcriptomic data from public databases, and although dataset-level batch effects were corrected, the potential influence of platform differences, cohort composition, and sample heterogeneity may persist. In particular, the discovery datasets included peripheral blood and synovial tissue. Tissue origin represents a biological source of variation rather than a purely technical batch effect, and the current public-data design did not allow a balanced tissue-stratified sensitivity analysis. Therefore, some DEGs, model features, and network signals may partly reflect tissue-specific expression patterns. Second, the single-cell dataset GSE200815 contains PsA rather than healthy controls as the comparator group. Therefore, the single-cell results should be interpreted as RA-vs-PsA differences and cannot support claims of RA-specific cellular changes relative to healthy individuals; they were used only for cellular localization and exploratory virtual perturbation. Third, although internal cross-validation-based resampling and independent external validation were used for model assessment, complete clinical-model validation was not performed. The internal validation procedure was designed to compare candidate algorithms and reduce direct information leakage, but it does not substitute for a prospectively prespecified diagnostic-model validation study. Calibration metrics such as Brier score, calibration slope, and calibration intercept were not available in the current analysis, and the model should not be regarded as a clinically validated diagnostic tool. Fourth, no experimental validation was performed in the present study. Virtual gene knockout results require validation in in vitro cell models or animal models, for instance by knocking down *HLA-DRB1* using small interfering RNA (siRNA) or clustered regularly interspaced short palindromic repeats (CRISPR) technology and observing effects on cell behavior, signaling pathways, and responses to aspartame stimulation. Fifth, molecular docking demonstrated a plausible binding pose between aspartame and *HLA-DRB1*; however, the binding affinity, binding kinetics, and impact on *HLA-DRB1* functions such as antigen binding, peptide-groove occupancy, and surface expression need to be determined through biophysical techniques such as surface plasmon resonance or isothermal titration calorimetry. Sixth, reverse causation and confounding cannot be excluded: inflammatory disease status, altered diet, medication use, or metabolic changes may influence sweetener exposure or metabolism rather than aspartame directly driving RA. Seventh, although the validation cohorts were not used during discovery or model construction, the initial restriction to RA DEGs and predicted aspartame targets may introduce feature-selection circularity and inflate apparent model performance; therefore, the RF + plsRglm model should be interpreted as a hypothesis-generating feature-prioritization model rather than a clinically validated diagnostic classifier without prospective validation.

The practical consequence is that tissue-enriched immune or stromal signals may affect both feature-importance rankings and network topology. Accordingly, the present model should not be interpreted as identifying tissue-independent RA biomarkers unless replicated in tissue-matched cohorts. Similarly, the reported AUCs quantify retrospective discrimination in public datasets and should not be taken as evidence of transportable clinical performance without prespecified probability calibration and prospective validation.

## 6. Conclusions

In summary, this study employed integrative bioinformatics approaches encompassing network toxicology, machine learning, single-cell analysis, and virtual gene knockout to identify a hypothesis-generating association between predicted aspartame targets and RA-related molecular networks. *HLA-DRB1* emerged as a prioritized immune-module candidate within a broader multigene network that also included inflammatory, protease/ECM-remodeling, adhesion, metabolic, and proliferation-related genes. The RA/PsA single-cell dataset provided cellular-context information rather than RA-versus-healthy-control validation, and the mixed tissue origin of the discovery datasets should be considered when interpreting the results. Molecular docking results suggested a structurally possible interaction between aspartame and *HLA-DRB1*. These findings provide a basis for further mechanistic investigation. Future studies should integrate prospective exposure data, independent cohorts with appropriate healthy controls, tissue-stratified or tissue-specific validation, biophysical binding assays, and in vitro and in vivo experiments to clarify whether the predicted aspartame-HLA-DRB1/RA relationship has biological and clinical relevance.

## Figures and Tables

**Table 1:** Top predicted gene perturbations after *HLA-DRB1* virtual knockout.

Gene	Distance	Z	FC	*p*.value	*p*.adj
*LUM*	8.33 × 10^−6^	2.569769162	6549.655307	0	0
*MMP2*	4.53 × 10^−6^	2.460897369	1941.393095	0	0
*AEBP1*	3.26 × 10^−6^	2.401960786	1000.785614	1.21 × 10^−219^	3.03 × 10^−216^
*BGN*	1.82 × 10^−6^	2.299646342	314.4635032	2.33 × 10^−70^	4.66 × 10^−67^
*COL6A3*	5.73 × 10^−7^	2.097451701	31.03371119	2.54 × 10^−8^	4.23 × 10^−5^
*PDGFRA*	4.45 × 10^−7^	2.053656679	18.6993202	1.53 × 10^−5^	0.021862434

**Note:** distance, network distance; Z, corrected network distance; FC, fold change (larger FC values indicate a greater impact of gene knockout on expression); *p*.value, nominal *p* value; *p*.adj, adjusted *p* value.

## Data Availability

All transcriptomic and single-cell datasets used in this study are publicly available from the Gene Expression Omnibus (GEO) database under the accessions listed in Section 4. Compound and protein information was obtained from the public databases described in the Methods. Further inquiries can be directed to the corresponding author.
